# Supplementary material for: Transcriptome Analysis Unravels Metabolic and Molecular Pathways Related to Fruit Sac Granulation in a Late-Ripening Navel Orange (Citrus sinensis Osbeck)
Source: Plants (Basel). 2020 Jan 11;9(1):95. doi: 10.3390/plants9010095 (PMC7020443; doi:10.3390/plants9010095)
Supplement: Supplementary file 1 [file plants-09-00095-s001.zip › plants-660528-supplementary(Table S1-S6, Fig. S1).doc]

**Appendix S**

**Table S1.** Comparison of mean temperature in January and extreme cold temperature in January

| year | Mean monthly temperature /°C | | | Average of the lowest daily temperature in January /°C | Average of January extreme cold temperature /°C | Days with low temperature ≤ 0 ℃ in January /d |
| --- | --- | --- | --- | --- | --- | --- |
|  | Jan. | Feb. | Dec. |  |  |  |
| 2008 | 1.5 | 5.1 | 7.8 | -0.4 | -4.6 | 21 |
| 2009 | 4.8 | 8.7 | 6.5 | 1.6 | -2.6 | 6 |
| 2010 | 5.5 | 6.7 | 8.7 | 3.0 | -1.6 | 3 |
| 2011 | 1.5 | 7.5 | 6.1 | -1.0 | -4.0 | 22 |
| 2012 | 4.1 | 4.9 | 5.2 | 2.2 | -1.8 | 7 |
| 2013 | 5.3 | 6.8 | 7.3 | 1.0 | -3.0 | 2 |
| 2014 | 7.5 | 5.5 | 7.1 | 4.0 | 1.0 | 0 |
| 2015 | 7.1 | 8.0 | 7.4 | 3.9 | -1.0 | 4 |
| 2016 | 4.9 | 9.4 | 8.9 | 2.9 | -4.8 | 4 |
| 2017 | 7.2 | 8.2 | 8.0 | 4.9 | -0.1 | 1 |
| 2018 | 3.7 | 7.2 | - | 1.3 | -4.2 | 11 |
| 2001  -2018 | 4.8 | 7.1 | 7.2 | 2.0 | - | - |

**Table S2.** Correlation analysis of low temperature in winter and fruit granulation of late navel orange

| Correlation coefficient | Average of the lowest daily temperature in January /°C | Average of January extreme cold temperature /°C | Days with low temperature ≤ 0 ℃ in January /d | Fruit granulation rate /% |
| --- | --- | --- | --- | --- |
| Average of the lowest daily temperature in January /°C | 1 | 0.7129* | -0.8251* | -0.8019* |
| Average of January extreme cold temperature /°C | 0.7129* | 1 | -0.5832 | -0.9314** |
| Days with low temperature ≤ 0 °C in January /d | -0.8251* | -0.5832 | 1 | 0.7745* |
| Fruit granulation rate /% | -0.8019* | -0.9314** | 0.7745* | 1 |

**Table S3** Investigation and comparison of fruit granulation of late navel orange at different altitudes and different harvest periods

| days after flowering /DAF | different altitude /m | | | | | | | | |
| --- | --- | --- | --- | --- | --- | --- | --- | --- | --- |
|  | 520 | | | 380 | | | 220 | | |
|  | granulation fruit numbers | research fruit numbers | granulation late /% | Granulation  fruit numbers | research fruit numbers | granulation late /% | granulation  fruit  numbers | research fruit numbers | granulation late /% |
| 235 | 0 | 30 | 0.0 | 0 | 30 | 0.0 | 0 | 30 | 0.0 |
| 250 | 0 | 30 | 0.0 | 0 | 30 | 0.0 | 0 | 30 | 0.0 |
| 265 | 0 | 30 | 0.0 | 0 | 30 | 0.0 | 0 | 30 | 0.0 |
| 300 | 0 | 30 | 0.0 | 0 | 30 | 0.0 | 0 | 30 | 0.0 |
| 335 | 1 | 30 | 3.3 | 0 | 30 | 0.0 | 0 | 30 | 0.0 |
| 350 | 6 | 40 | 15.0 | 0 | 30 | 0.0 | 0 | 30 | 0.0 |
| 365 | 19 | 38 | 50.0 | 0 | 30 | 0.0 | 0 | 30 | 0.0 |
| 400 | 43 | 105 | 41.0 | 1 | 30 | 3.3 | 0 | 30 | 0.0 |

**Table S4.** Contrast of measurement of winter and spring field average temperature and January extreme low temperature at different altitudes

| The altitude/m | Mean monthly temperature /°C | | | Average of the lowest daily temperature in January /°C | Average of the lowest daily temperature in February /°C | Extreme cold temperature in January and February /°C |
| --- | --- | --- | --- | --- | --- | --- |
|  | Dec. | Jan. | Feb. |  |  |  |
| 220 | 7.8 | 7.0 | 5.5 | 3.6 | 2.9 | -2.0 |
| 380 | 6.9 | 5.9 | 4.5 | 2.5 | 1.8 | -2.8 |
| 520 | 5.8 | 5.1 | 3.8 | 2.0 | 1.0 | -4.1 |

**Table S5.** Overview of the data obtained by RNA-seq

| Constructed library | GR | | CK | |
| --- | --- | --- | --- | --- |
|  | Repeat1 | Repea2 | Repeat1 | Repea2 |
| Total raw reads | 12,262,850 | 11,765,449 | 12,240,220 | 12,994,442 |
| Total clean reads | 12,175,100  (99.28%) | 11,648,841  (99.01%) | 12,109,834 (98.93%) | 12,842,648  (98.83%) |
| Reads mapped to genes | 9,206,118  (75.61%) | 8,699,914  (74.68%) | 9,138,778  (75.47%) | 9,750,063  (75.92%) |
| perfect match to genes | 6,959,979  (57.17%) | 6,550,344  (56.23%) | 6,854,467  (56.60%) | 7,341,529  (57.17%) |
| Reads mapped to genome | 10,033,399  (82.41%) | 9,587,519  (82.30%) | 9,951,209  (82.17%) | 10,575,502  (82.35%) |
| Perfect match to genome | 7,466,725  (61.33%) | 7,106,771  (61.01%) | 7,348,538  (60.68%) | 7,839,681  (61.04%) |
| Multi-position match | 712,293  (5.85%) | 684,754  (5.88%) | 718,110  (5.93%) | 760,582  (5.92%) |

**Table S6.** Information of primers for qPCR

| **Gene ID** | **Gene name** | **primers (5’-3’)** | |
| --- | --- | --- | --- |
|  |  | **Forward Primer** | **Reverse Primer** |
| *Cs*1g17210 | Protein TIFY 10A | GCCTCTCTCCAACGTTTCTT | CCTGGATACTTGCCTTGATCTT |
| *Cs*1g19920 | Cell wall protein PRY3 | GACTACGACATGAAGGGTTCAG | GAGTGTAATGGCCGCACATA |
| *Cs*4g13870 | Oxidase | AATGGCCCTTCTGCATCAA | ACCAGTTGCTTCACCTTCTC |
| *Cs*5g31400 | Myc-like regulatory protein | GATGTGTGTTTCCTTCTCGTTTC | ACCTCATTTGCTCCGGTAAG |
| *Cs*5g12420 | Glycoprotein EP1 | TTCAAGGTGCTCCCGAATG | ACTGACCCACCAAGAGAGTA |
| *Cs*6g21320 | Amino acid transporter | ACTCTGTACACTTCCATGAAGAA | CAGAACCAAACATCATGTACCC |
| *Cs*9g06490 | Carboxylesterase | CGATAGTGCAGGAGCTAACAT | CCAGAAGTACGGGTGAGTTAAA |
| *Cs*3g22560 | Sucrose transport protein | TCAGCGTTCCGTTTGCTATG | CCAGCATCTGTGGAACAACTAT |
| *Cs*7g08770 | Galactinol synthase | GCTGAGCAAGATTTCCTGAATATG | TTCTCAAGCTCCACGTTCTC |
| *Cs*7g31410 | Intrinsic protein | CCAGAGACTCTCATGTTCCTATTT | TTGATGCCAGTTCCGGTAATA |
| *Cs*8g04610 | GH3 family protein | GACTTCGGCTGGTGAAAGAAA | GTCCAAGCCAGGCACATAAA |
| *Cs*1g21210 | Aminotransferase | GTGCAATTTACTCCAACGATGA | GAGCAAAGCGGAGAGAAGATAC |
| *Cs*5g22170 | Protein kinase | GATTGGCTTGGGCAGTTTG | TCGCATCATCTAGCACCTTATT |
| *Cs*8g06820 | Mannitol transporter | GAGCCAAGAGCAACAACAATAC | CCGGAGGCATACAGATTTCTT |
| *Cs*5g01960 | Cellulose synthase | TGAGGACTACTACACTGGGTATC | TTGGTGCATCGCCGTAAA |
| orange1.1t00214 | Pectin methylesterase | GGTACAGCCCAGAAGAAGTAAA | CAGTGTCTTGTTTGATGGAAGTG |
| *Cs*2g07660 | Pectin methylesterase | GCATGGATTTGACCATCATCAC | CCATCACCACCAACAGCTATC |
| *Cs*2g31410 | Alcohol acyl transferase | CACTCAAGCTACAACCTGAAGA | CCACAATAGCCTGGAGGAATATC |
| *Cs*1g15830 | Auxin-responsive protein | TCCCGAGCAAGACTGTTAATG | AGGGACACCATCCATGTTAATC |
| *Cs*7g08620 | Polygalacturonase | CTCCTCTCTCTCTTCTCCTTCT | ACGTCGCCGGAAGTATTG |
| *Cs*7g18970 | Polygalacturonase-  inhibiting protein | ACATGCGATCTCACCACTAAC | CGAGGTAAGGAAGATCACCAAC |
| *Cs*7g01980 | Polygalacturonase-  inhibiting protein | GTCCTCACTCAACGAACCTTAC | GACTCACCACGAAGCGAAATA |
| *Cs*6g21420 | Calcium-binding protein | CCAGGTTGTTTGAGGAGAAAGA | GCAACTCCAGGGCATCAATA |
| *Cs*1g16550 | pectin methylesterase | TTCCGGCCAAAAAAATATGG | TGAATGACAATGCCGGTGTT |
| *Cs*1g12840 | Polygalacturonase | GACATGCTCCAACGGCTATT | GACTTCAACACCACGAGATGC |
| *Cs*5g20320 | Cellulase | CTGGGCTCTCTGATGGGTTATTTG | AGGATAGCATTGTTGTCGTGAAGG |
| *Cs*8g16290 | Phenylalanine ammonia-lyase | TTATGGGTTCAAAGGTGCCG | GTTGCTCAGCACTTTGGACG |
| *Cs*1g04910 | Cinnamyl-alcohol dehydrogenase | GATTTGCCACTCGGACTTGC | CAGGAGGCTACCAAGACCCC |
| orange1.1t02040 | Peroxidase | GACAGCAGAACAGCCAACAGG | GTTCGGCACTGAGCCCTTC |
| *Cs*1g05000 | Actin | CCATCCAGGCTGTTCTATCTC | GGAAGGGCATATCCCTCATAAA |

**Figure S1.** Quality assessment of reads
